# Fiber stiffness, pore size and adhesion control migratory phenotype of MDA-MB-231 cells in collagen gels

**DOI:** 10.1371/journal.pone.0225215

**Published:** 2019-11-13

**Authors:** Florian Geiger, Daniel Rüdiger, Stefan Zahler, Hanna Engelke

**Affiliations:** 1 Department of Chemistry and Center for NanoScience, Ludwig-Maximilians-Universität München, Munich, Germany; 2 Department of Pharmacy, Ludwig-Maximilians-Universität München, Munich, Germany; Seoul National University College of Pharmacy, REPUBLIC OF KOREA

## Abstract

Cancer cell migration is influenced by cellular phenotype and behavior as well as by the mechanical and chemical properties of the environment. Furthermore, many cancer cells show plasticity of their phenotype and adapt it to the properties of the environment. Here, we study the influence of fiber stiffness, confinement, and adhesion properties on cancer cell migration in porous collagen gels. Collagen gels with soft fibers abrogate migration and promote a round, non-invasive phenotype. Stiffer collagen fibers are inherently more adhesive and lead to the existence of an adhesive phenotype and in general confined migration due to adhesion. Addition of TGF-β lowers adhesion, eliminates the adhesive phenotype and increases the amount of highly motile amoeboid phenotypes. Highest migration speeds and longest displacements are achieved in stiff collagen fibers in pores of about cell size by amoeboid phenotypes. This elucidates the influence of the mechanical properties of collagen gels on phenotype and subsequently migration and shows that stiff fibers, cell sized pores, and low adhesion, are optimal conditions for an amoeboid phenotype and efficient migration.

## Introduction

Migration of cancer cells is a complex process. It is influenced by properties of the migrating cells as well as their environment [1]. While the environmental properties, such as stiffness, size, and density as well as spatial distribution of adhesion sites, are well controllable for migration on two-dimensional, continuous substrates, these properties are significantly more complex in three-dimensional porous hydrogels [2]. Consequently, migration in two dimensions is fairly well understood, but the influence of environmental parameters on three-dimensional migration still remains to be understood [1, 3]. Key parameters of the environment that influence migration in three dimensions are confinement, adhesion sites and stiffness [4, 5]. Strong confinement below a certain threshold can stall migration entirely, since it blocks movement of the nucleus [6]. The influence of adhesion sites depends on cell phenotype. Some migration phenotypes are dependent on force transmission via adhesion, while other phenotypes are independent of adhesion [7–9]. Adding to the complexity is the fact, that cells can switch migratory phenotype not only due to biochemical stimuli, but also depending on the environmental properties [10–14]. Since the phenotype strongly impacts migratory properties, this switching behavior influences migration significantly and impacts speed, persistence and other parameters. In complex heterogeneous environments such plasticity may promote invasion [15]. On the other hand, cells can remodel their environment and thus influence its properties that impact migration [16, 17]. A prominent example is matrix degradation via enzymes, which allows for directed movement in strongly confined matrices [18, 19]. The dependence of three-dimensional migration on cell phenotype and the environment with the mutual feedback interactions opens a huge phase space of influences on three-dimensional migration [1]. Here, we investigated the influence of the mechanical properties of hydrogels on migratory phenotype and with that on migration properties. Hydrogel remodeling of cells in our study was minimal. This allowed us to analyze the influences of environment on cell phenotype without further complexity due to changes in the environment induced by cell remodeling. Confinement and adhesion have been shown to significantly influence migratory phenotype and migration parameters in continuous substrates [10]. Yet, tissue is highly heterogeneous and parts of it resemble porous collagen meshworks rather than continuous glass or plastic channels [20–22] and the difference between continuous and porous substrates may significantly alter migration properties [23]. Here, we show that fiber stiffness, confinement and adhesion behavior of the cell strongly influenced migratory phenotype and with that migration parameters in porous hydrogels. While some influences resembled those in continuous substrates, we also found strong differences.

## Results

**Fig 1:**
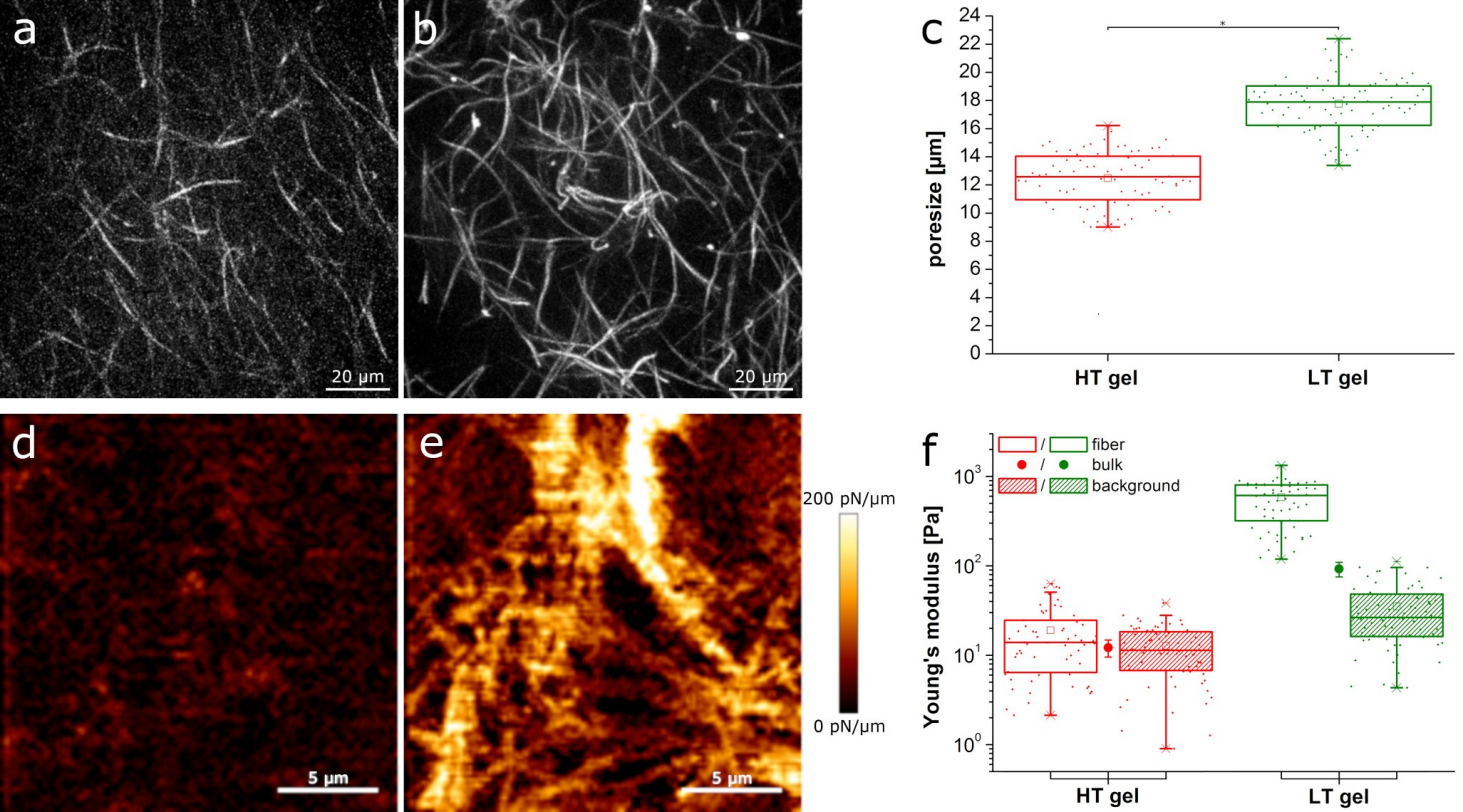
Collagen gel characterization. a) Fluorescence image of an HT gel polymerized at 37°C and b) of a LT gel polymerized at lower temperatures; c) pore sizes of HT and LT gels respectively (p<0.001, two-sample t-test); AFM image proportional to the local stiffness of d) an HT and e) an LT gel; f) Young’s modulus of fibers and background as measured by AFM and Young’s modulus of the bulk gel as measured with a rheometer for HT and LT gels respectively.

To investigate the influence of different local hydrogel structures on cell migration, we prepared two different types of collagen gels. Both gel types consisted of 1.85 mg/ml rat tail collagen. The first type of gel (HT) was polymerized instantly at 37°C. The other was first cooled on ice and then polymerized at room temperature (LT). The polymerization at different temperatures created gels differing in pore size as well as fiber thickness. Fig 1A shows the fine structure of the HT gels with thin fibers and small pore sizes of 12.5 μm on average. The LT gels in Fig 1B consist of thicker fibers and larger pore sizes of about 18 μm on average (Fig 1C). The cell size is on average 15.5 ± 5.5 μm in HT gels and 13.4 ± 4.8 μm in LT gels. Both gel types however contain pores that are comparable to the size of cells. Thus, we can exclude that cells are stuck due to pore sizes that do not allow for penetration of the cell nucleus as described in [6].

While the overall concentration of collagen and with that the amount of adhesion sites is the same for both types of gels, the local amount of adhesion sites on collagen fibers will be higher on the thicker fibers of the LT gels [24]. The difference in fiber thickness will also affect fiber stiffness and with that local compliance of the gel. We measured fiber stiffness of the gels using atomic force microscopy and the resulting maps of the slope of the force-distance curves that are proportional to the local stiffness are displayed in Fig 1D and 1E. While LT gels show clearly visible, stiff fibers, the stiffness of HT fibers is barely above background. Quantitatively, we obtained an average Young’s modulus of 19 ± 16 Pa for HT fibers with a background of 12.7 ± 7.6 Pa and a Young’s modulus of more than an order of magnitude higher of 584 ± 296 Pa with a background of 35 ± 26 Pa for LT gels (Fig 1F). Thus, the fiber to background ratio of HT gels is only about 1.5, while it is 16.6 for LT gels. Bulk measurements on a rheometer revealed a Young’s modulus of 12.8 ± 2.5 Pa for HT gels and 82 ± 14 Pa for LT gels in accordance with literature [25].

**Fig 2:**
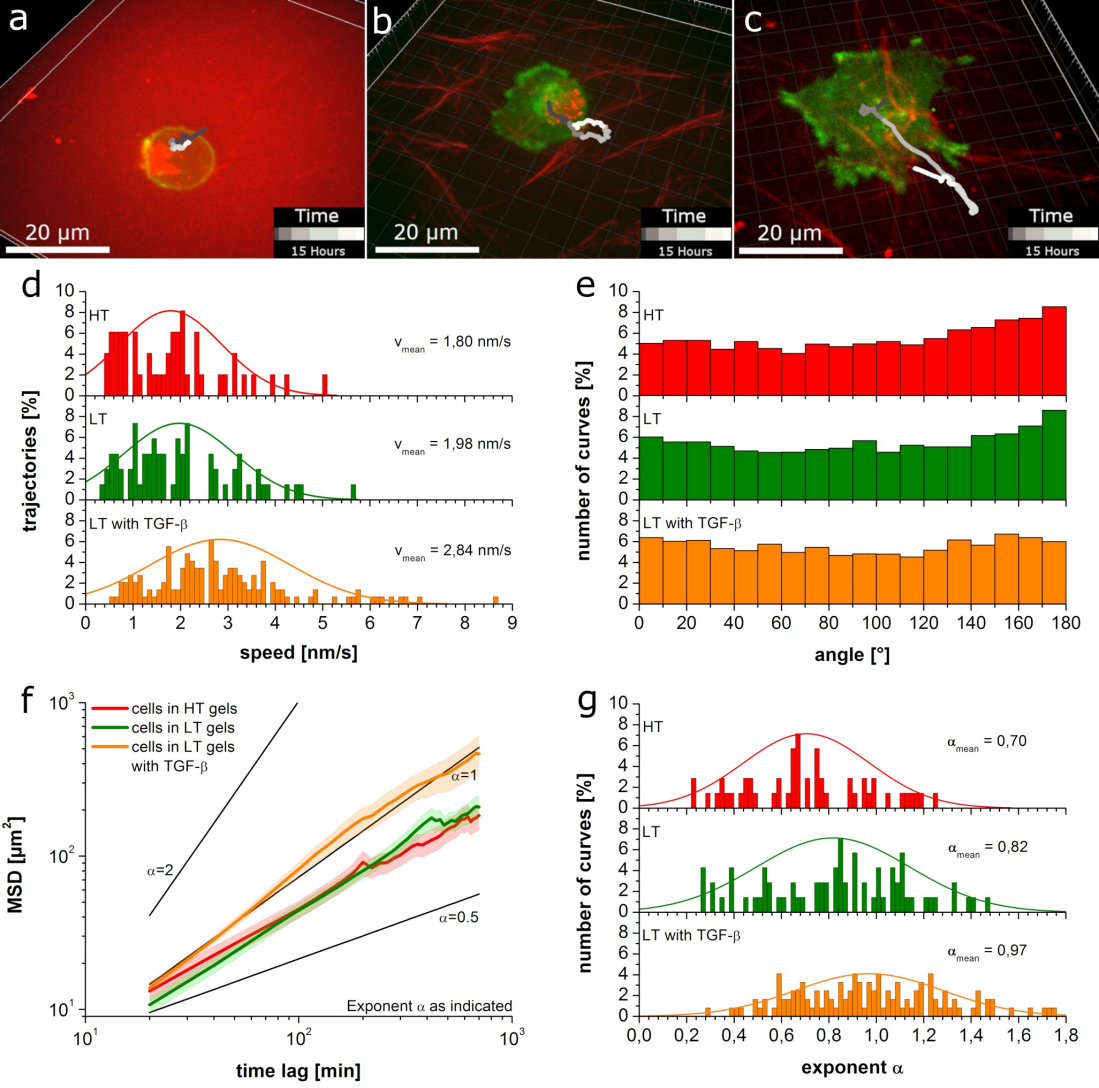
MDA-MB-231 migration in collagen gels. a)-c) representative images of cells (green) and their migratory tracks (gray shades) in HT gels (a), LT gels (b) and LT gels in presence of TGF-β (c) respectively (collagen fibers in red); images represent 3D xyz-stacks at the first measured time point and trajectories (in gray shades) represent the 3D path over time with a time scale as indicated in the color bar in the lower right corners; d) histogram of speed distributions show the increase in speed upon addition of TGF-β; e) angle distribution between different steps shows significant backtracking due to confinement for HT and LT gels (HT compared to LT: p < 0.005, HT compared to LT with TGF-β: p < 0.001, LT compared to LT with TGF-β: p < 0.001); f) average mean square displacements for the different conditions show subdiffusive behavior in the absence of TGF-β; g) histogram of exponent α values show the increase of α upon addition of TGF-β.

Next, we analyzed migration of MDA-MB-231 cells in fluorescently labeled LT and HT gels. MDA-MB-231 is an invasive breast cancer cell line. They migrate in various phenotypes and adapt their phenotype and migration behavior flexibly depending on the prevalent conditions [26–28]. Cells were labeled with lifeAct-GFP marking filamentous actin within the cell. Representative images of cells and their migration tracks imaged every 20 min over 15 h are displayed in Fig 2A–2C. Cells in HT gels (Fig 2A) show very small displacements and seem stuck at their initial position. In LT gels, cells explore a larger space over time, yet the observed movement is still very limited (Fig 2B). Thus, we added TGF-β to cells in LT gels. TGF-β is a drug that is known to promote cell migration. Indeed, upon addition of TGF-β we find cells to be more motile with a significant amount of cells moving through pores of the collagen matrix yielding fairly straight trajectories (Fig 2C). Addition of TGF-β to cells in HT gels on the other hand, did not lead to any visible changes in migration. We did not observe any significant matrix degradation or remodeling in any of the experiments except for occasional small deformations of the matrix resulting from cells pushing themselves through pores of the matrix or pushing fiber ends out of their way while passing.

Quantitative analysis revealed a distribution of cell speed as displayed in Fig 2D (see also S1 Fig for statistical analysis and S2 Fig for HT gels with TGF-β). While cells move with a similarly low average speed in HT gels and LT gels, their speed distribution is clearly shifted to higher speeds upon addition of TGF-β in LT gels, but not in HT gels. The distribution of angles (Fig 2E and S2 Fig) between steps in LT and HT gels shows a tendency toward backtracking, i.e. 180° turns, which may result from confined movement for example due to pore size or strong adhesion [29]. Migration tracks in the presence of TGF-β show a fairly uniform angle distribution resembling that of a random walk. Also the average mean squared displacement (MSD) showed an increase in slope upon addition of TGF-β to LT gels confirming the decrease in confinement (Fig 2F). Fitting the MSD of each single trajectory to a random walk equation and accounting for anomalous diffusion with the exponent α, we obtained a distribution of the exponent α as shown in Fig 2G (see also S1 Fig for statistical analysis and S2 Fig for HT gels with TGF-β). Values of α around 1.0 reveal diffusive behavior and values below 1.0 subdiffusive behavior, e.g. due to confinement. Values above 1.0 indicate superdiffusive behavior, which is typical for cells migrating with persistence for example through channels or on surfaces. In HT gels the exponent α is below 1 with very few exceptions, indicating subdiffusive behavior for most of the cells independent of TGF-β. In LT gels without drug, α is slightly higher with more cells (33%) reaching values above 1. However, on average, most of the cells still show subdiffusive behavior. In the presence of TGF-β in LT gels more than 43% of the cells reach α values above 1.0.

Taken together, these results show confined migration for both gel types. This confinement is removed upon addition of TGF-β to LT gels. This drug is known to activate RhoA signaling [30], which favors myosin driven contractility over actin polymerization and thus less adhesive amoeboid migration modes over adhesive mesenchymal migration [31, 32].

**Fig 3:**
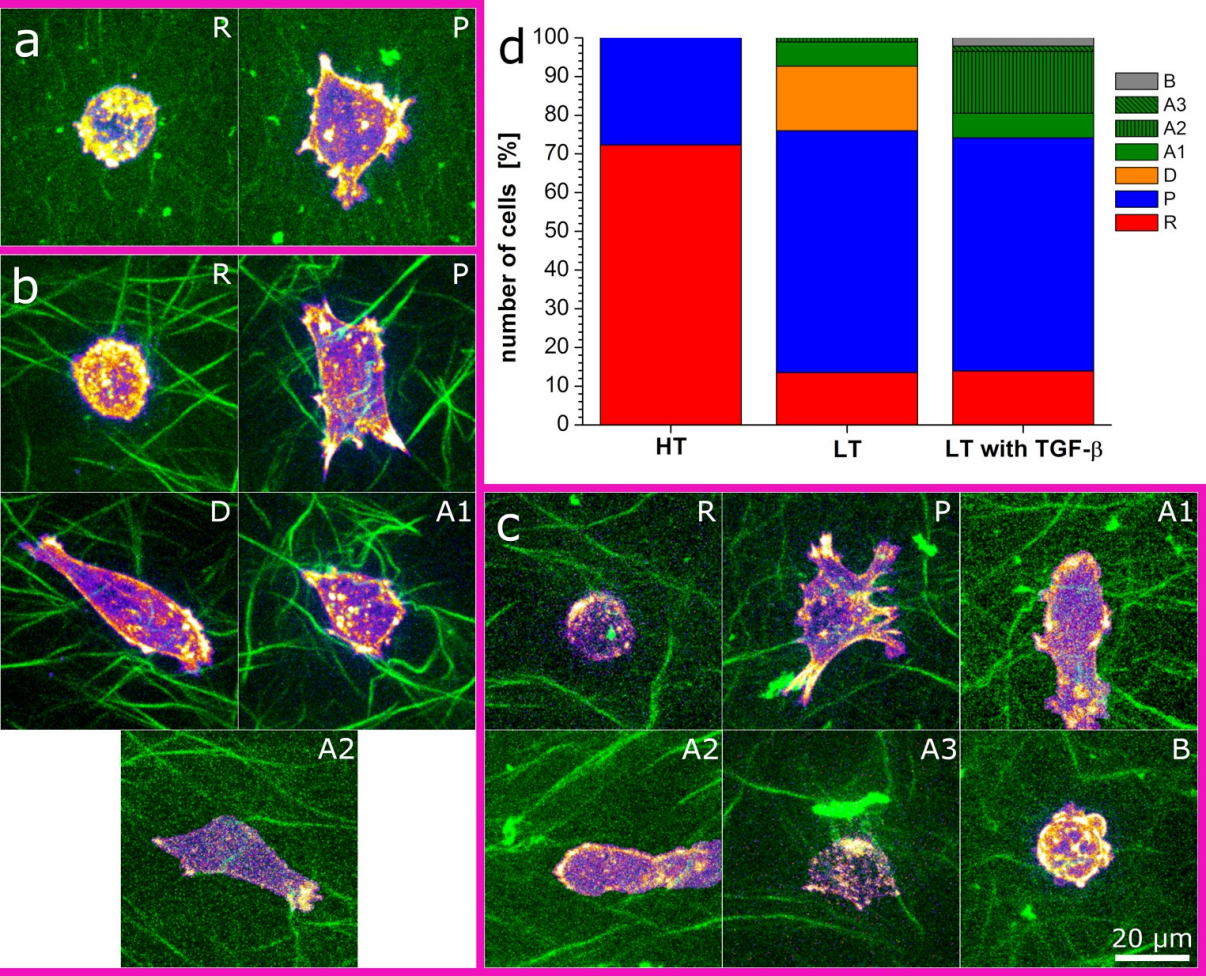
Migratory phenotypes of MDA-MB-231 cells in collagen gels. a) round (R) and pseudopodial (P) phenotype in HT gels; b) round (R), pseudopodial (P), adherent (D), amoeboid, including ellipsoid with leading edge (A1) and squeeze (A2) phenotype in LT gels, c) round (R), pseudopodial (P), amoeboid including ellipsoid with leading edge (A1), squeezing (A2), and rear driven (A3), and blebbing (B) phenotype in LT gels upon addition of TGF-β; collagen fibers are displayed in green and cellular actin is shown in purple (low concentration) and yellow (high concentration) d) phenotype distribution within the different gel types showing the high amount of round cells in HT gels, the adherent cells specifically occurring in LT gels and the increase in amoeboid cells in LT gels upon addition of TGF-β.

To analyze the influence of local environment and TGF-β on cell migration, we thus determined the cell phenotypes (Fig 3A–3D and S3 Fig). In HT gels we find exclusively round cells and cells with pseudopods. Round cells represent the majority in HT gels without TGF-β. In LT gels cells with pseudopods are the majority and only about 10% show a round phenotype. Additionally, we find a spread, adherent phenotype and few cells show amoeboid character. Addition of TGF-β to LT gels enhances the amount of amoeboid cells and it removes the adherent, spread phenotype entirely. The amoeboid cells show several subforms, namely a movement with a fairly round or ellipsoid cell body and a leading edge, a movement consisting of squeezing through pores with a dented cell body, and occasionally the rear driven movement described for MDA-MB-231 cells in matrigel by Poincloux et al. [33]. Very few cells showed a blebbing phenotype.

**Fig 4:**
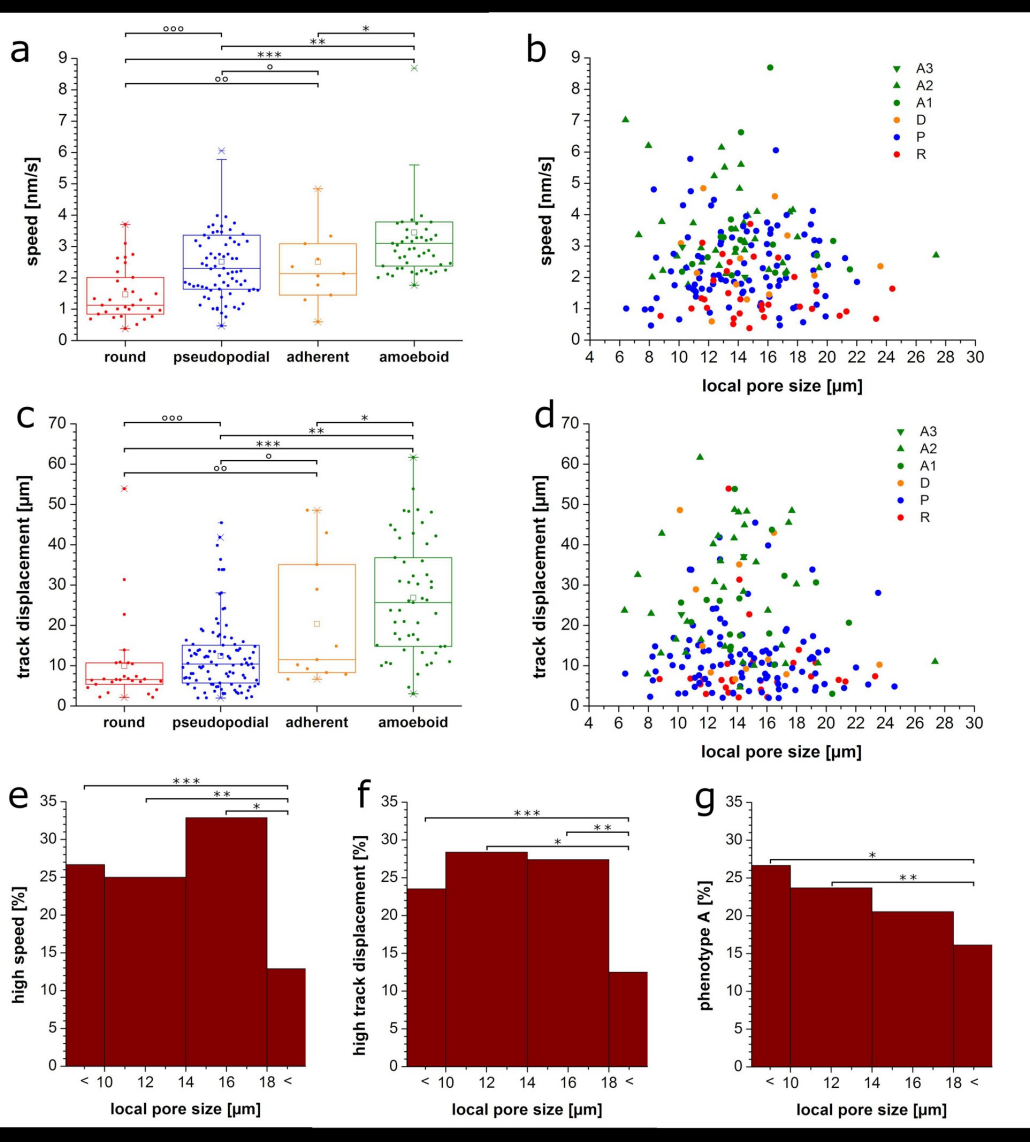
Migration parameters depending on phenotype and pore size. a) speeds for different phenotypes, p < 0.001 (*p < 0.02,**p < 0.001, ***p < 0.001,°p < 0.95,°°p < 0.02,°°°p < 0.001); b) speed versus local pore size for different phenotypes; c) track displacement for different phenotypes, p < 0.001,(*p < 0.1, **p < 0.001, ***p < 0.001,°p < 0.2,°°p < 0.005,°°°p < 0.02); d) track displacement versus local pore size for different phenotypes including round (R), pseudopodial (P), adherent (D), and amoeboid with the subforms ellipsoid with leading edge (A1), squeezing (A2), and rear driven (A3) cells; e) fraction of cells in respective pore size with speed values greater than 3.2 nm/s (0.75 percentile of all cells), 4 μm binning, (*p < 0.01, **p < 0.1, ***p < 0.06); f) fraction of cells in respective pore size with track displacement values greater than 22.8 nm/s (0.75 percentile of all cells), 4 μm binning, (*p < 0.05, **p < 0.05, ***p < 0.1); g) fraction of cells in respective pore size with amoeboid phenotype, 4 μm binning, (*p < 0.1, **p < 0.1).

Analysis of the motility parameters for the observed migration phenotypes shows that the amoeboid phenotype is fastest and least confined leading to high α-values and the longest track displacements, i.e. end-to-end-distances (Fig 4A–4D and S4 Fig). The round phenotype on the other hand is the slowest (Fig 4A) and most confined with low α-values (S4 Fig) and very short track displacements (Fig 4C). Thus, the altered phenotype distribution combined with an observed increase in motility of pseudopodial cells might explain the enhanced average cell motility upon addition of TGF-β to LT gels.

Further analysis of the influence of the local pore size at the cell location on phenotype and migration parameters reveals pore sizes much larger than cell size to impede migration leading to a lower fraction of fast cells and a lower fraction of high track displacements (Fig 4B and 4D–4G, fast speed and high displacement cells being defined as cells with the respective value higher than 75% of all cells). Also the amount of cells with amoeboid phenotype is reduced at large pore sizes. At the same time this phenotype reaches highest speeds, α-values and track displacements (Fig 4A and 4C and S4 Fig). Looking at the maximum values in Fig 4B and 4D, also confirms that amoeboid cells at pores of around cell size reach highest speeds and longest track displacements. A round phenotype on the other hand can be found at all pore sizes with its relative amount increasing with pore size (S5 Fig). Its speed, α-value, and track displacement is low. The pseudopod phenotype occurs in all pore sizes with almost uniform distribution except for a small tendency toward pores smaller than cell size (S5 Fig).

## Discussion

Taken together, HT gels impede migration. Although their pore size is in a range that does not obstruct migration and the low amount of adhesion sites on the thin HT gel fibers should promote amoeboid migration, these gels entirely abrogate migration even in the presence of TGF-β. Thus, the missing compliance due to the low fiber stiffness of HT gels supposedly inhibits migration. The missing fiber stiffness blocks force transmission along the fibers independent of the cell behavior. Hence, sufficient fiber stiffness is a prerequisite of migration and reducing it, a save measure to inhibit migration independent of cell type. This encourages therapeutic efforts that target the extracellular matrix stiffness, which otherwise increases with age and cancer progression [34]. Interestingly, the low fiber stiffness does not only impair movement itself, but also prevents establishment of motile amoeboid phenotypes suggesting that cells react to the stiffness [35] and that motile phenotypes result from a mechanically coupled feedback as discussed in [1]. While pore size and fiber stiffness of LT gels allow for migration, the high amount of adhesion sites favors adhesive phenotypes of low motility, which is removed by lowering adhesion with TGF-β. Highest speeds and longest track displacements are achieved at pores that are no larger than a cell. Hence, low adhesion, pores, which are no larger than cell size, and comparably high fiber stiffness offers best conditions for migration. Here, the fiber stiffness allows for good force transmission and low adhesion prevents confinement due to long lasting focal adhesions [24]. At the same time low adhesion does not allow for force transmission via integrins. This necessitates the pore sizes, which are not much larger than cell size. They allow for chimneying movements as described by Malawista et al [36] where the cell pushes itself along fibers [37]. Accordingly, we observe amoeboid migration mainly between two parallel fibers at a distance of about cell size and slightly smaller. This allows for fairly straight movement along the parallel fibers (see S1 Movie) without the necessity of reorientation. At the same time this distance allows for easy movement without barriers for the nucleus to pass. The requirement of low adhesion and confinement for a switch to an amoeboid phenotype is in accordance to movement in continuous substrates [10, 13].

Other than in continuous substrates, where round cells were found under no confinement and low adhesion [10], in the porous meshwork of collagen gels studied here, round cells occurred under all confinements. Yet, their proportion increased with increasing pore size in LT gels. They were also the prevalent phenotype in migration inhibiting HT gels without TGF-β. This suggests that they arise from the absence of a mechanical compliance, such as adhesion, confinement or stiffness. Mechanically non-compliant environments lack cues for a symmetry break, thus the cell stays in the symmetric round phenotype. In other words, the absence of compliance in HT gels favors a round phenotype, the porous, non-continuous meshwork in LT gels that provide less mechanical compliance than continuous substrates support round phenotypes less than HT gels, yet still in almost all situations, and the highly compliant continuous substrates allow for the round phenotype only in the least compliant situation of low adhesion and low confinement. Finally, while the blebbing state was reported to be the transition state in homogenous, continuous substrates [10], in porous collagen networks, the pseudopodial state is the state, in which cells reside most often in LT gels and from which they switch to other phenotypes. This might be due to the inhomogeneous meshwork, within which cells have to probe the different directions with pseudopods before transitioning into the most favored phenotype. Probing of the different spatial directions of course is not necessary in homogenous substrates.

In conclusion we show the plasticity of MDA-MB-231 cells in porous hydrogels. Even small differences of biochemical or mechanical stimuli can have huge impact on migratory phenotype and resulting migration properties.

## Materials and methods

### Cell culture

MDA-MB-231 were obtained from ATCC (ATCC® HTB-26^™^) and cultured in Dulbecco’s modified Eagle’s medium (DMEM, Gibco), supplemented with 10% fetal bovine serum (FBS, Gibco) and 1% Penicillin Streptomycin (Gibco), at 37°C in a 5% CO_2_ atmosphere. Cells were regularly checked to be mycoplasm free with PCR Myoplasma Test Kit I/C (PromoKine). To image cells, their actin was labeled with LifeAct-TagRFP (Ibidi) or LifeAct-TagGFP2 (Ibidi). To stably express LifeAct-proteins, selection was performed using 50 ng/ml of G418 (Geneticin).

### 3D collagen matrices

Collagen gels were prepared with ice-cooled compounds. Rat tail collagen I stock solution (Corning, high concentration) was mixed with DMEM-Medium (45% of final volume), containing 100000 cells/ml and neutralized with Sodium hydroxide (1 N, Fluka). The mixture was diluted with Dulbecco's phosphate-buffered saline (DPBS(1x), Gibco) to a final collagen concentration of 1.85 mg/ml. To label the fibers of the gel either 0.5% w of the collagen was replaced by fluorescein isothiocyanate (FITC) conjugated type I collagen (AnaSpec) or the collagen was mixed with 1 μg ATTO-633 (NHS-Ester, ATTO-TEC) per 2.74 mg collagen. If indicated TGF-β was added to the mixture to a final concentration of 26.7 ng/ml. After preparation the gel mixture was immediately filled into a sample carrier (Nunc^™^ Lab-Tek^™^ II, 8 wells) on ice for the gelation process.

For HT gels, the gelation process was at 37°C. For LT gels, the sample is kept on ice for 30 minutes, followed by 15 minutes at room temperature and finally at 37°C for at least about 30 minutes. After the gelation all gels were overlaid with DMEM.

### Spinning disk microscopy

Microscopy for live-cell imaging was performed on a Zeiss Cell Observer SD with a Yokogawa spinning disk unit CSU-X1. The objective was a 1.40 NA 63x Plan apochromat oil immersion objective from Zeiss. Measurements were performed at 37°C and a 5% CO_2_ humidified atmosphere. FITC-collagen and LifeAct-TagGFP2 were imaged using a 488 nm laser, ATTO-633 with a 639 nm laser and LifeAct-TagRFP with a 561 nm laser. For two colour detection of FITC-collagen and LifeAct-TagRFP fusion protein or LifeAct-TagGFP2 fusion protein and ATTO-633, a dichroic mirror (660nm, Semrock) and band-pass filters 525/50 and 690/60 (both Semrock) were used in the detection path. Separate images for each fluorescence channel were acquired using two separate electron multiplier charge coupled devices (EMCCD) cameras (PhotometricsEvolve^TM^). Time-lapse images were acquired with a frame time of 20 min and 50 frames.

### Rheology

Bulk rheology was performed on a MCR 100 rheometer (Anton Paar) and gels were prepared directly on the rheometer. The chemicals and preparation was the same as described above. 400 μl were filled between a PP25 measuring plate and a thermostatic plate. After 48 min 400 μl of water were spread around the polymerizing gel to prevent drying out. The gap size during gelation was 0.6 mm and was reduced to 0.5 mm during the measurement. The deformation was measured stepwise up to a final deformation of 10% at 37°C and a frequency of 1 Hz.

Stiffness of the fibers was measured with the AFM NanoWizard® 4 (JPK Instruments) and SPM software (JPK Instruments) with an integrated Axiovert 200 inverted microscope (Zeiss). Collagen gels were prepared as described above and 100 μl gel were distributed in a 35mm round glas dish (MatTek). Briefly, V-shaped cantilevers (Bruker; MLCT-D silicon nitride, resonance frequency 15 kHz, spring constant 0.03 N/m) were calibrated with the contact free method and used in the QI^™^ Mode (Advanced Imaging). The following values have been set: setpoint 0.2–0.4 nN, z-length 5 μm; speed 80 μm/s and pixel size 128x128 on a 20x20 μm grid. During the measurement the height and the slope of the force-distance curves were recorded. Data processing was performed using the corresponding software version 6.0.50 (JPK Instruments). After median filtering, background substraction and a low pass filter, stiffness was calculated on representative data points using the Hertzian contact model (Young’s modulus). The tip shape was modeled as quadratic pyramid, the half-front angle of the cantilever as 15° and its Poisson ratio was set to 0,5.

### Image analysis

Analysis of pore and cell size was performed with ImageJ employing BoneJ [38–40]. Migration trajectories and related properties were analyzed with Imaris (v 8.2.0, Bitplane, AG Zurich, Switzerland) and further processed with custom-written code in Octave (version 4.2.0). Statistics and data presentation was done with OriginPro (Version 8.0891, OriginLab Corporation, Northampton, MA, USA). Phenotypes were analyzed by visual inspection. Normality was tested with a Shapiro-Wilk test and depending on the outcome, a two-sample t-test (pore size of gels, indicated in the respective figure caption) or a non-parametric test (all other cases) was used. In this case, Kruskal-Wallis Anova tests were used for tests with more than two groups and Mann-Whitney Tests for comparison of two groups. For statistical tests of differences between different fractions as a function of pore size, binomial tests were performed. Boxplots show mean (square), the box consisting of median, lower and upper quartile (25 th and 75 th percentile), whiskers (5th and 95th percentile), and outliers (marked x).

Speed was calculated as the square root of the MSD at *τ* = 0 time lag divided by the frame time; angles from the distribution are calculated from the cosine between two successive steps of the trajectory; track displacement is calculated as the distance between first and last position of the center of mass of the cell; the exponent α is determined via a fit to the first 5 lag times of the MSD according to the equation *MSD(τ) = D·τ*^*α*^ with D and α as free fit parameters. Cells with fast speed and high track displacement respectively, were defined as cells showing the respective value higher than 75% of all cells independent of pore size.

## Supporting information

S1 Fig Statistical analysis of MDA-MB-231 migration in collagen gels. a) box plots of speed distributions show statistical differences between LT with TGF-β and the gels without, but no difference between the two gels without TGF-β (*p < 0.001, **p < 0.001, ***p < 0.49); b) box plots of exponent α values show a significant increase of α upon addition of TGF-β to LT gels, as well as a significant difference between LT and HT gels (*p < 0.002, **p < 0.001, ***p < 0.05).
(TIFF)

S2 Fig MDA-MB-231 migration in collagen gels–addition of TGF-β to HT gels. a) histograms of speed distributions show no increase in speed upon addition of TGF-β to HT gels (HT compared to HT with TGF-β, p < 0.6); b) angle distribution between different steps shows significant backtracking due to confinement for HT gels, which is removed upon addition of TGF-β (HT compared to HT with TGF-β, p< 0.001, HT with TGF-β compared to LT with TGF-β, p < 0.4) c) average mean square displacements for the different conditions show subdiffusive behavior in HT gels independent of TGF-β; d) histograms of exponent α values show no increase of α upon addition of TGF-β to HT gels (HT compared to HT with TGF-β, p < 0.4).
(TIFF)

S3 Fig Phenotype distribution in HT gels and both gels with TGF-β. In HT gels only round and pseudopodial phenotypes were observed (with and without TGF-β), while LT gels with TGF-β show a wider variety of phenotypes including amoeboid cells.
(TIFF)

S4 Fig Distribution of α-values depending on phenotype. a) α-values for different phenotypes show an enhanced α-value for amoeboid cells, p < 0.01 (*p < 0.17, **p < 0.01, ***p < 0.001,°p < 0.83,°°p < 0.95,°°°p < 0.28); b) α-values versus local pore size for different phenotypes including round (R), pseudopodial (P), adherent (D), and amoeboid with the subforms ellipsoid with leading edge (A1), squeezing (A2)), and rear driven (A3) cells; c) fraction of cells in respective pore size with alpha values greater than 1.14 (0.75 percentile of all cells), 2 μm binning, (*p < 0.08, **p < 0.07, ***p < 0.02,****p < 0.005); d) fraction of cells in respective pore size with alpha values greater than 1.14 (0.75 percentile of all cells), 4 μm binning, (*p < 0.08, **p < 0.07); e) fraction of cells in respective pore size with speed values greater than 3.2 nm/s (0.75 percentile of all cells), 2 μm binning, (*p < 0.008, **p < 0.03, ***p < 0.05, ****p < 0.06); f) fraction of cells in respective pore size with track displacement values greater than 22.8 nm/s (0.75 percentile of all cells), 2 μm binning, (*p < 0.08, **p < 0.008, ***p < 0.03).
(TIFF)

S5 Fig Fraction of phenotypes depending on pore size. a) fraction of cells in respective pore size with pseudopodial phenotype, 4 μm binning, (*p < 0.01, **p < 0.02, ***p < 0.1); b) fraction of cells in respective pore size with round phenotype, 4 μm binning, (*p < 0.1, **p < 0.09).
(TIFF)

S1 Movie MDA-MB-231 cell with amoeboid phenotype migrating in LT gel. (AVI)

S2 Movie MDA-MB-231 cell with round phenotype migrating in LT gel. (AVI)

S3 Movie MDA-MB-231 cell with pseudopodial phenotype migrating in LT gel. (AVI)
