# Supplementary figures and images for: Fiber stiffness, pore size and adhesion control migratory phenotype of MDA-MB-231 cells in collagen gels
Source: PLoS One. 2019 Nov 13;14(11):e0225215. doi: 10.1371/journal.pone.0225215 (PMC6853323; doi:10.1371/journal.pone.0225215)

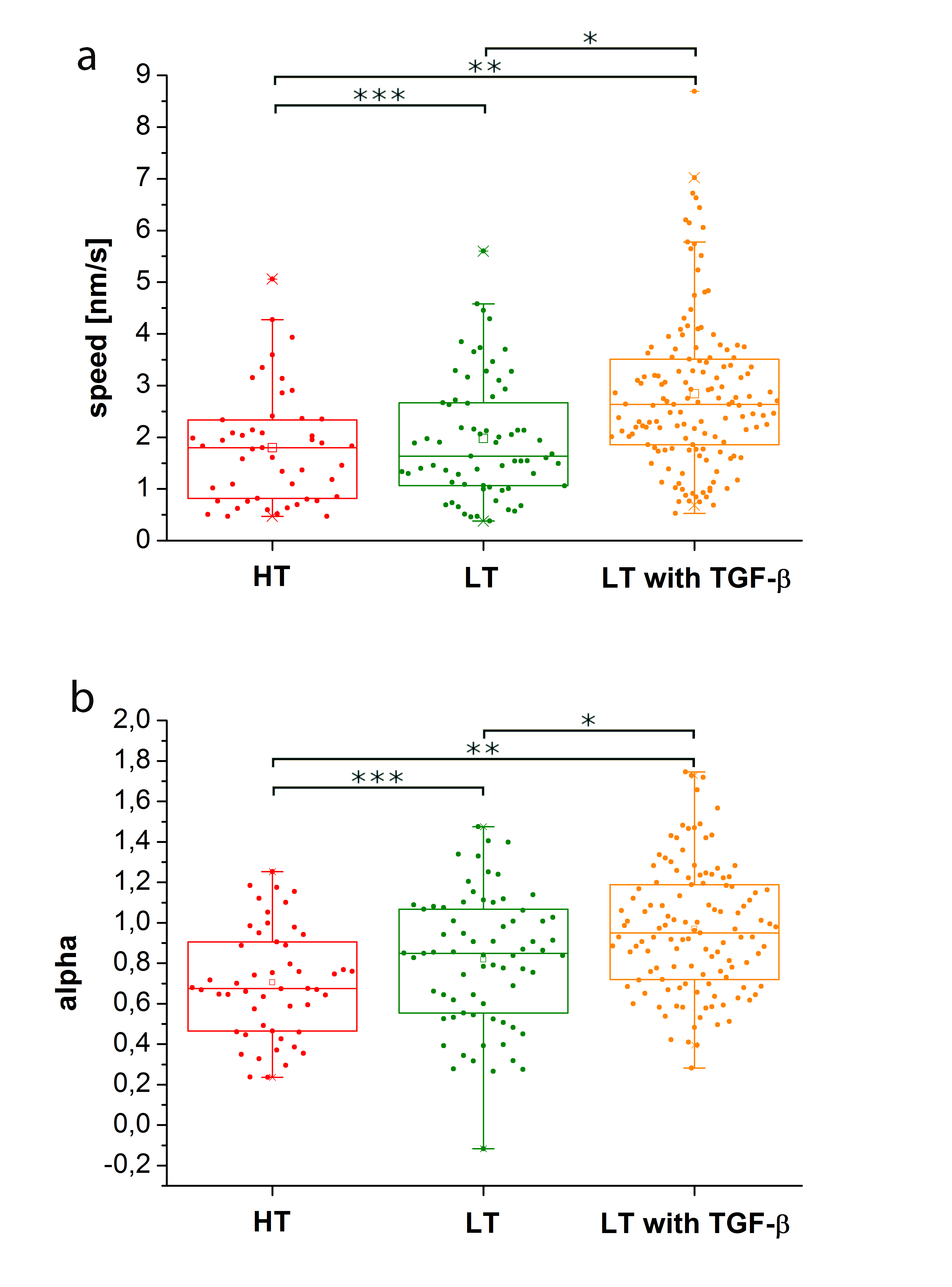

Supplement: S1 Fig — a) box plots of speed distributions show statistical differences between LT with TGF-β and the gels without, but no difference between the two gels without TGF-β (*p < 0.001, **p < 0.001, ***p < 0.49); b) box plots of exponent α values show a significant increase of α upon addition of TGF-β to LT gels, as well as a significant difference between LT and HT gels (*p < 0.002, **p < 0.001, ***p < 0.05). (TIFF) [file pone.0225215.s001.tiff]

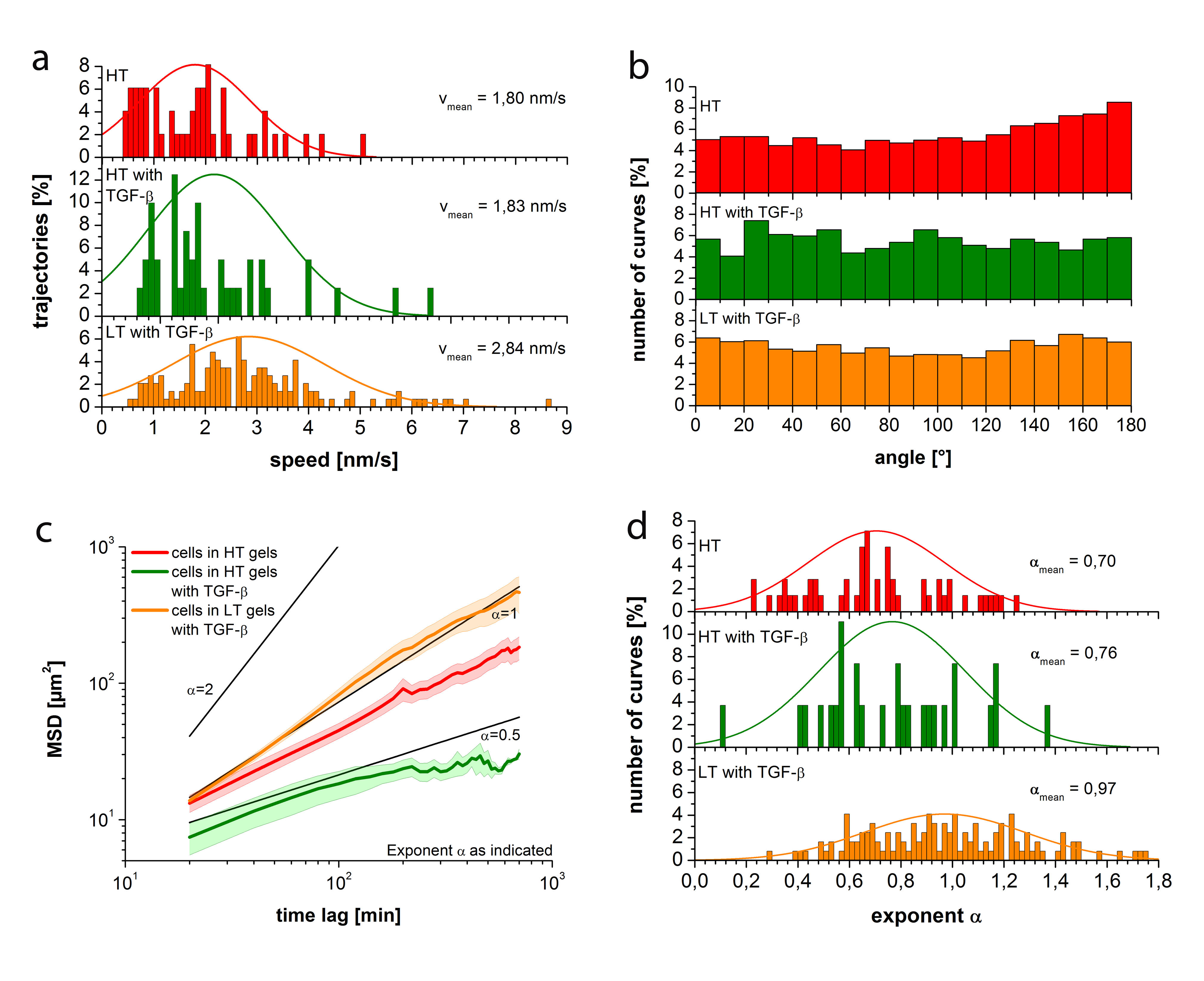

Supplement: S2 Fig — a) histograms of speed distributions show no increase in speed upon addition of TGF-β to HT gels (HT compared to HT with TGF-β, p < 0.6); b) angle distribution between different steps shows significant backtracking due to confinement for HT gels, which is removed upon addition of TGF-β (HT compared to HT with TGF-β, p< 0.001, HT with TGF-β compared to LT with TGF-β, p < 0.4) c) average mean square displacements for the different conditions show subdiffusive behavior in HT gels independent of TGF-β; d) histograms of exponent α values show no increase of α upon addition of TGF-β to HT gels (HT compared to HT with TGF-β, p < 0.4). (TIFF) [file pone.0225215.s002.tiff]

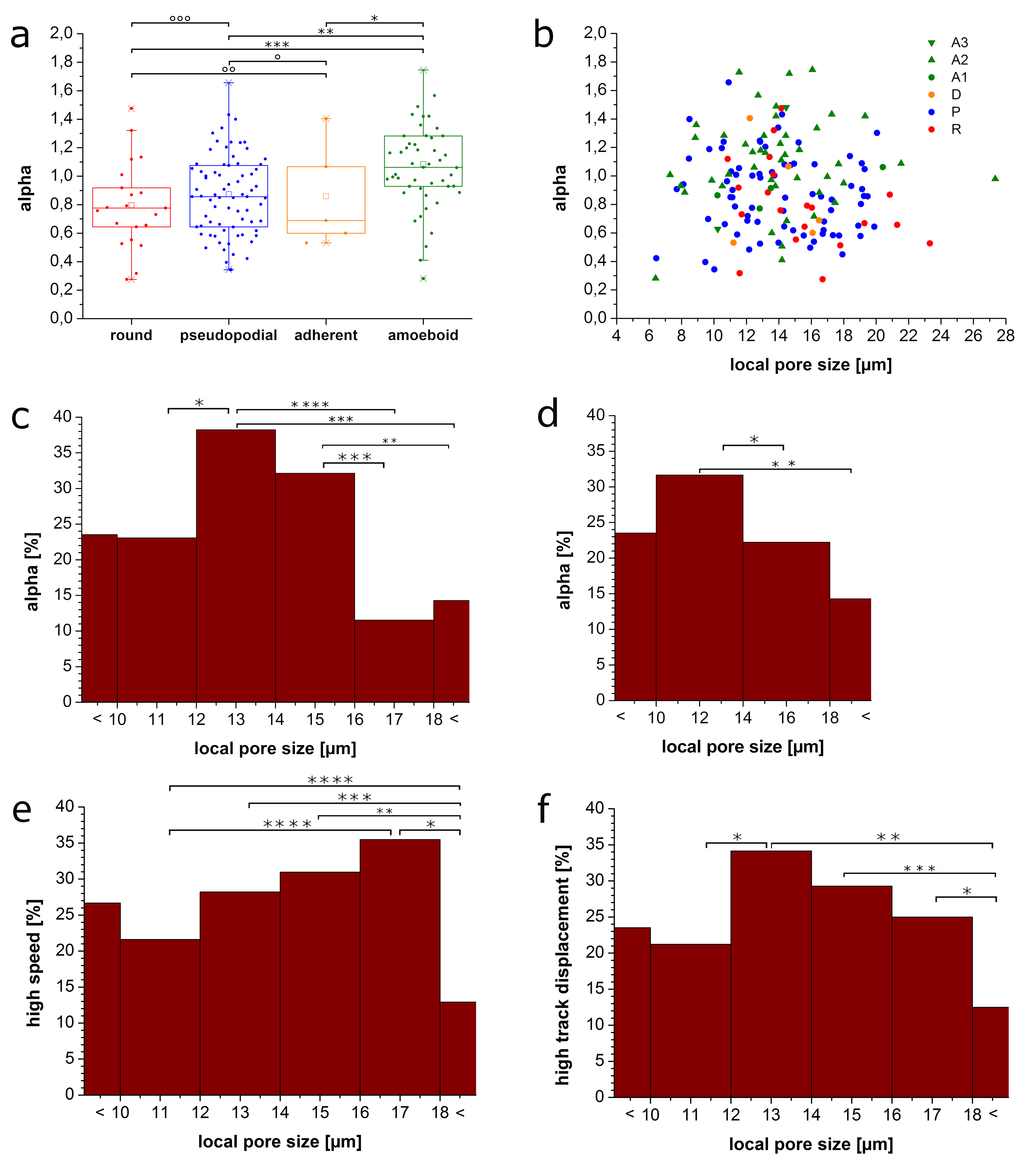

Supplement: S4 Fig — a) α-values for different phenotypes show an enhanced α-value for amoeboid cells, p < 0.01 (*p < 0.17, **p < 0.01, ***p < 0.001,°p < 0.83,°°p < 0.95,°°°p < 0.28); b) α-values versus local pore size for different phenotypes including round (R), pseudopodial (P), adherent (D), and amoeboid with the subforms ellipsoid with leading edge (A1), squeezing (A2)), and rear driven (A3) cells; c) fraction of cells in respective pore size with alpha values greater than 1.14 (0.75 percentile of all cells), 2 μm binning, (*p < 0.08, **p < 0.07, ***p < 0.02,****p < 0.005); d) fraction of cells in respective pore size with alpha values greater than 1.14 (0.75 percentile of all cells), 4 μm binning, (*p < 0.08, **p < 0.07); e) fraction of cells in respective pore size with speed values greater than 3.2 nm/s (0.75 percentile of all cells), 2 μm binning, (*p < 0.008, **p < 0.03, ***p < 0.05, ****p < 0.06); f) fraction of cells in respective pore size with track displacement values greater than 22.8 nm/s (0.75 percentile of all cells), 2 μm binning, (*p < 0.08, **p < 0.008, ***p < 0.03). (TIFF) [file pone.0225215.s004.tiff]

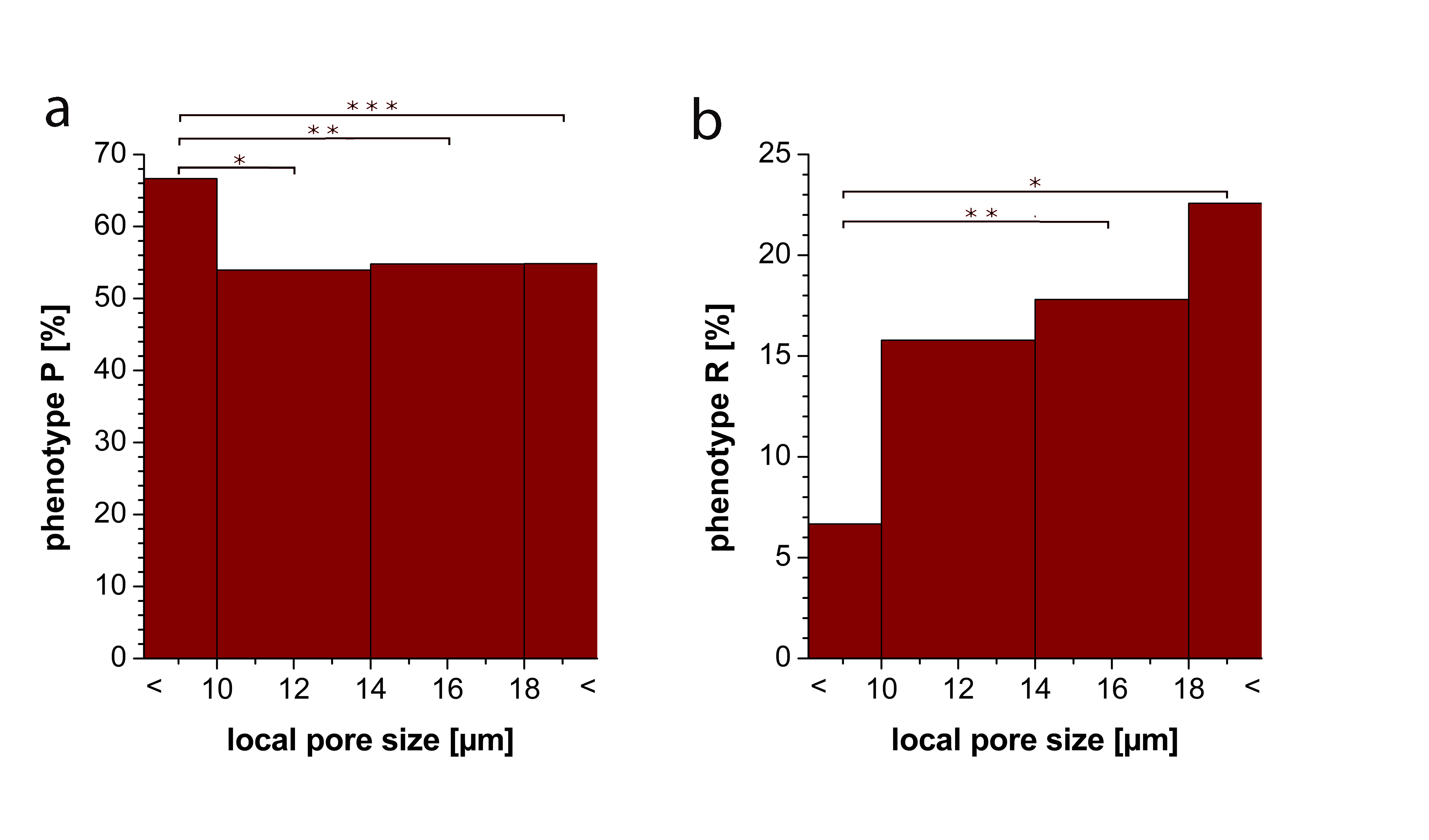

Supplement: S5 Fig — a) fraction of cells in respective pore size with pseudopodial phenotype, 4 μm binning, (*p < 0.01, **p < 0.02, ***p < 0.1); b) fraction of cells in respective pore size with round phenotype, 4 μm binning, (*p < 0.1, **p < 0.09). (TIFF) [file pone.0225215.s005.tiff]
